# Human Coronavirus NL63 Open Reading Frame 3 encodes a virion-incorporated N-glycosylated membrane protein

**DOI:** 10.1186/1743-422X-7-6

**Published:** 2010-01-15

**Authors:** Marcel A Müller, Lia van der Hoek, Daniel Voss, Oliver Bader, Dörte Lehmann, Axel R Schulz, Stephan Kallies, Tasnim Suliman, Burtram C Fielding, Christian Drosten, Matthias Niedrig

**Affiliations:** 1 University of Bonn Medical Centre, Sigmund-Freud-Str. 25, D-53127 Bonn, Germany; 2 Robert Koch-Institut, Center for Biological Safety, Nordufer 20, D-13353 Berlin, Germany; 3 University of Amsterdam, Laboratory of Experimental Virology, Center for Infection and Immunity Amsterdam (CINIMA), Academic Medical Center, Meibergdreef 15, 1105 AZ, Amsterdam, The Netherlands; 4 University of the Western Cape, Department of Medical Biosciences, Private Bag X17 Bellville 7535, Republic of South Africa

## Abstract

**Background:**

Human pathogenic coronavirus NL63 (hCoV-NL63) is a group 1 (alpha) coronavirus commonly associated with respiratory tract infections. In addition to known non-structural and structural proteins all coronaviruses have one or more accessory proteins whose functions are mostly unknown. Our study focuses on hCoV-NL63 open reading frame 3 (ORF 3) which is a highly conserved accessory protein among coronaviruses.

**Results:**

*In-silico *analysis of the 225 amino acid sequence of hCoV-NL63 ORF 3 predicted a triple membrane-spanning protein. Expression in infected CaCo-2 and LLC-MK2 cells was confirmed by immunofluorescence and Western blot analysis. The protein was detected within the endoplasmatic reticulum/Golgi intermediate compartment (ERGIC) where coronavirus assembly and budding takes place. Subcellular localization studies using recombinant ORF 3 protein transfected in Huh-7 cells revealed occurrence in ERGIC, Golgi- and lysosomal compartments. By fluorescence microscopy of differently tagged envelope (E), membrane (M) and nucleocapsid (N) proteins it was shown that ORF 3 protein colocalizes extensively with E and M within the ERGIC. Using N-terminally FLAG-tagged ORF 3 protein and an antiserum specific to the C-terminus we verified the proposed topology of an extracellular N-terminus and a cytosolic C-terminus. By *in-vitro *translation analysis and subsequent endoglycosidase H digestion we showed that ORF 3 protein is N-glycosylated at the N-terminus. Analysis of purified viral particles revealed that ORF 3 protein is incorporated into virions and is therefore an additional structural protein.

**Conclusions:**

This study is the first extensive expression analysis of a group 1 hCoV-ORF 3 protein. We give evidence that ORF 3 protein is a structural N-glycosylated and virion-incorporated protein.

## Background

The human Coronavirus (hCoV)-NL63 constitutes one of four circulating prototypic human Coronaviruses (CoV) [1]. HCoV-NL63 infection causes upper and lower respiratory tract disease and is globally widespread, particularly among children under the age of six years [2-4]. It was shown to be associated with croup [5,6].

CoV belong to the *Nidovirales*. The CoV genome consists of a 27 to 33 kb positive single-stranded RNA which is 5'-capped and 3'-polyadenylated [7]. The genome of hCoV-NL63 comprises 27,553 nt and has a gene organization conserved in all CoV, i.e., gene 1a/b, spike (S), open reading frame 3 (ORF 3), envelope (E), membrane (M) and the nucleocapsid (N) gene. CoV virions consist of a nucleocapsid core surrounded by an envelope containing three membrane proteins, S, E, and M. CoV assemble and bud at membranes of the endoplasmic reticulum (ER)-Golgi intermediate compartment (ERGIC) [8,9]. While the budding site of several CoV has been localized at the ERGIC, the viral surface proteins can also be found in downstream compartments of the secretory pathway [8]. M localizes predominantly in the Golgi apparatus [10,11], S is found along the secretory pathway and at the plasma membrane [12,13], and E is detected in perinuclear regions, the ER and Golgi [14-16]. S and M are typically glycosylated and it was shown that glycosylation plays an important role in the generation of bioactive protein conformations and influences fusion activity, receptor binding, and antigenic properties of CoV [17-20].

In addition to the S, E, M and N protein genes, the structural gene portion of CoV genomes contains a variable number of accessory ORFs. Because these accessory ORFs are not shared between different CoV groups, they are also referred to as group-specific ORFs [21]. Proteins encoded by group-specific ORFs of different CoV have been shown to influence pathogenesis, virus replication, or host immune response [21-27]. Others may be dispensable for virus replication in cultured cells of primate or rodent origin, as well as in rodent models [26,28,29].

The ORF 3 is the only accessory ORF conserved in all CoVs [30]. Most investigations of its functionality have been done on the example of SARS-CoV ORF 3a. The SARS-CoV ORF 3a protein is expressed in infected cells and patient sera contained antibodies reactive with recombinant ORF 3a antigen. The N-terminal ectodomain was able to induce virus-neutralizing antibodies in rabbits [31]. SARS-CoV ORF 3a protein is a triple-spanning membrane protein with a similar topology as the M protein, and is integrated into virions [32]. Moreover, truncated forms were discovered for recombinantly and virally expressed ORF 3a protein which could also be detected in virions [33]. Unlike the M protein it is not N-glycosylated but O-glycosylated and it was shown to interact with E, M and S protein [16,34-36]. Subcellular localization of ORF 3a protein was found to be at the Golgi complex and the plasma membrane where it was also internalized by endocytosis [36]. ORF 3a protein was shown to induce apoptosis [37] and cell cycle arrest [38] and to up-regulate expression of fibrinogen in lung epithelial cells [39]. Although small interfering RNAs targeting the ORF 3a-specific viral subgenomic RNA were able to reduce viral replication [40], deletion of ORF 3a from an infectious cDNA clone had no effect on viral replication in cell culture and mice [28]. Moreover it has been demonstrated that SARS-ORF 3a protein forms a homotetramer through inter-protein disulfide bonds, functionally working as a potassium ion channel that modulates virus release [41]. Very recently it was shown that the ORF 3a protein disrupts the architecture of the Golgi apparatus and might thus be responsible for the formation of vesicular structures in which virus replication takes place [42].

SARS-CoV as a member of CoV group 2b (beta) is only distantly related to the human CoV-NL63, a member of group 1b (alpha). For the ORF 3 protein of group 1 (alpha) CoVs investigations have focused on the porcine epidemic diarrhea virus (PEDV, group 1b, alpha) and transmissible gastroenteritis virus (TGEV, group 1a, alpha) that cause enteropathogenic diarrhea in swine [43]. It was shown that virulence of these viruses could be reduced by altering the ORF 3 gene through cell culture adaptation [44,45]. For hCoV-NL63, preliminary experiments suggested that deletion of ORF 3 had little influence on viral replication in cell culture [46]. However, the closely related hCoV-229E has a homologous gene named ORF 4 that is split into two ORFs (4a and 4b) in cell culture but maintained in all circulating viruses. This suggests an *in-vivo *function that may not be necessary for viral replication in cell culture [47].

In the present study we characterized the ORF 3 protein of hCoV-NL63. We analyzed the expression and subcellular localization of the ORF 3 protein in virus-infected cells and cells transfected transiently with ORF 3 protein-expressing plasmids. We determined the topology of the ORF 3 protein, characterized its glycosylation, and showed that the ORF 3 protein is a structural protein incorporated into viral particles.

## Results and Discussion

**Figure 1:**
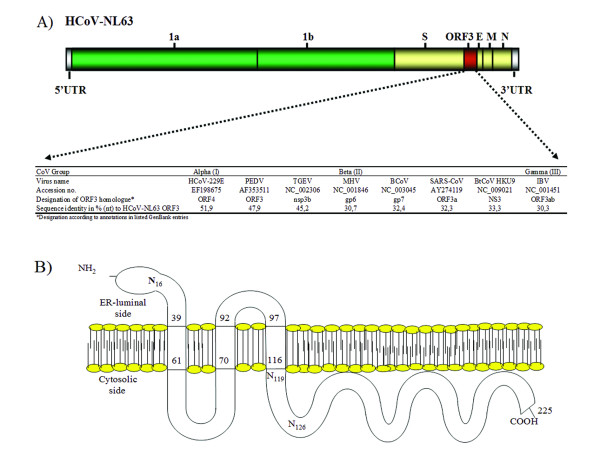
**Characteristics of hCoV-NL63 open reading frame 3 and comparison to homologous genes in other coronaviruses**. The sequence of ORF 3 (GenBank accession no. AY567487.2) was analyzed using BLAST and MEGA4. (A), localization of ORF 3 within the hCoV-NL63 genome and comparison of nucleotide (nt) identity based on multiple sequence alignments with prototype strains of CoV groups alpha, beta, gamma. Note that IBV ORF 3a and b were fused to one ORF 3ab. (B), Summarized results of *in-silico *analysis on membrane topology and glycosylation (refer to Materials and Methods section). Predicted N-linked glycosylation sites are indicated by an "N" at the respective localizations with an index number indentifying the amino acid position. No O linked glycosylation sites were predicted.

The hCoV-NL63 genome contains an open reading frame (ORF 3) situated between the S and E genes (Figure 1A). Nucleic acid sequence alignments with homologous genes of other CoV from groups alpha, beta and gamma yield nucleotide identities between 30,3% and 51,9% (Table within Figure 1A). Amino acid alignments showed highest levels of similarity (62%) and identity (43%) between hCoV-NL63 ORF 3 protein and the homologous protein of hCoV-229E [48]. A constant level of similarity was observed across the whole protein. *In-silico *analysis of potential glycosylation sites and membrane topology suggest properties similar to SARS-CoV ORF 3a protein (Figure 1B and Table 1). HCoV-NL63 encodes a 225 aa protein (approximately 26 kDa) with three putative transmembrane domains at aa positions 39-61, 70-92 and 97-116, respectively (TMHMM analysis). It has three potential N-glycosylation sites (NXS/T) at aa positions 16, 119 and 126, of which probably only the first is used because the sites at positions 119 and 126 are located inside the predicted transmembrane domains. No O-glycosylation sites are predicted. Nearly half of the protein (108 of 225 aa) forms a hydrophilic C-terminus. These findings are in concordance with earlier data comparing SARS-CoV 3a-like CoV proteins [35].

**Table 1:** Comparison of viral proteins ORF 3 and M of hCoV-NL63 and SARS-CoV^a^

Viral protein	hCoV-NL63 ORF 3	SARS-CoV ORF 3a	hCoV-NL63 M	SARS-CoV M
No. amino acids [size in kDa]	225 [26]	274 [31]	226 [26]	221 [25]

No. transmembrane domains (position)	3 (39-61, 70-92, 97-116)	3 (34-56, 77-99,103-125)	4 (20-38, 43-65, 75-97, 129-151)	3 (15-37, 50-72, 77-99)
No. cysteine residues (position)	4 (72, 131, 137, 182)	8 (81, 117, 121, 127, 130, 133, 148, 157)	4 (54, 67, 90, 180)	3 (158, 63, 85)
No. putative N-glycosylation sites (position)	3 (16, 119, 126)	1 (227^b^)	3 (3, 19, 188)	1 (4^c^)
No. putative O-glycosylation sites (position)	-	3 (28^c^, 32^c^, 267-271)	-	-

^a^Positions of aa refer to accession no. NC_005831 (hCoV-NL63) and AY278491 (SARS-CoV)

^b^Not used

^c^Usage confirmed

### Expression and subcellular localization of ORF 3 protein in virus-infected cells

**Figure 2:**
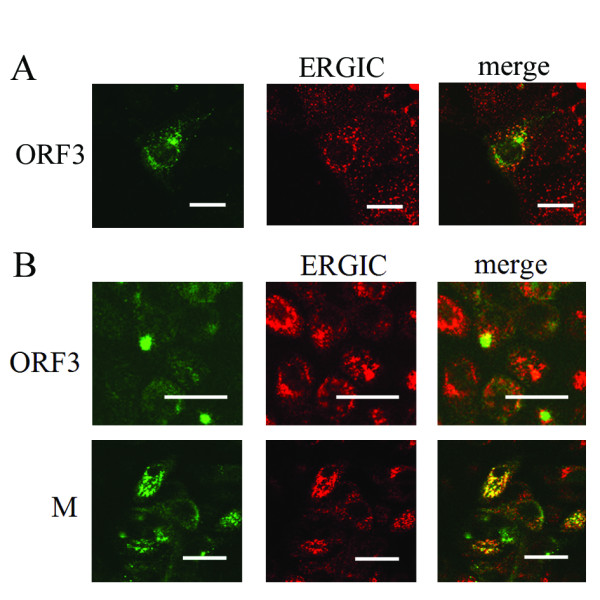
**Subcellular localization of viral proteins in hCoV-NL63 infected CaCo-2 and LLC-MK2 cells by immunofluorescence assay**. Confocal laser scanning microscopy on CaCo-2 (A) and LLC-MK2 cells (B) infected with hCoV-NL63. Left panels: staining with anti-ORF 3 and anti-M protein rabbit antisera (only in B) and detection by fluorescein isothiocyanate (FITC)-labelled goat-anti-rabbit antibody (green). Middle panels: detection of co-staining of the same cells with mouse-anti-ERGIC-53 mAB (Axxora) and detection with rhodamine-labelled goat-anti-mouse antibody. Yellow signals in merged pictures (right panels) show colocalization. Bars represent 20 μm.

To analyze the expression of ORF 3 protein during viral replication, colon carcinoma cells (CaCo-2) and Rhesus monkey kidney cells (LLC-MK2) cells were infected with hCoV-NL63 and an immunofluorescence assay (IFA) was done after two and four days, respectively. A rabbit polyclonal antiserum raised against a peptide representing the C-terminal aa 211-225 of the predicted ORF 3 protein yielded fluorescence in the cytoplasm as shown in Figure 2A and 2B (upper panel). Because colocalization of SARS-CoV ORF 3a protein with the ERGIC has been reported [36,49], the same cells were counterstained with a murine monoclonal antibody against the ERGIC53 marker protein. As shown in Figure 2A and 2B (upper panel) colocalization was observed in CaCo-2 and LLC-MK2 cells. Because overlapping subcellular localization was reported for SARS-CoV proteins 3a and M [50], it was analyzed whether hCoV-NL63 ORF 3 and M proteins were located in the same compartment. As shown in Figure 2B (bottom panel), a strong colocalization was also seen for anti-NL63 M and anti-ERGIC53 signals.

### Subcellular localization of transfected ORF 3 protein in human hepatocellular carcinoma cells (Huh-7) cells

**Figure 3:**
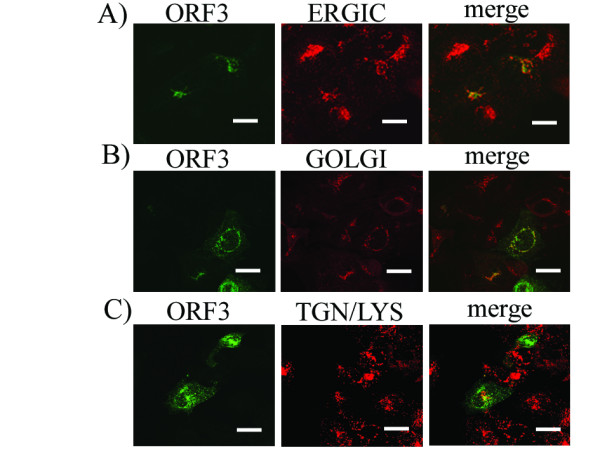
**Subcellular localization study of overexpressed hCoV-NL63 ORF 3 protein in Huh-7 cells**. Confocal laser scanning microscopy on cells expressing recombinant ORF 3 protein and co-staining with different antibodies for cellular organelles. Left panels: staining with rabbit-anti-ORF 3 serum and anti-rabbit-Cy2 (Dianova). Middle panels from top to bottom: co-staining of cellular organelles with a mouse-anti-ERGIC53 (A), mouse-anti-Golgi 58 K for the Golgi (B), goat-anti-LAMP-1 for *trans*-Golgi Network (TGN) and Lysosomes (LYS) together with goat (or donkey)-anti-mouse-Cy3 antibodies (C). Right panels show merged pictures where yellow areas represent colocalization. Partial colocalizations can be observed with all organelle markers indicating that the glycoprotein ORF 3 is processed *trans-*Golgi. Bars indicate 20 μm.

After showing that the ORF 3 protein can be found within the ERGIC compartment in infected cells we were interested in which other cellular compartments an isolated overexpressed ORF 3 protein can be detected. Therefore we transfected Huh-7 cells and stained the ORF 3 protein with the specific antiserum and co-stained different cellular compartments with specific antibodies (mouse-anti-ERGIC53, mouse-anti-Golgi 58 K, goat-anti-LAMP-1 for *trans*-Golgi/Lysosomes). As shown in Figure 3 the recombinant ORF 3 protein can be detected in all major compartments of the secretory pathway (Figure 3A for ERGIC, 3B for Golgi and 3C for *trans*-Golgi and lysosomes). These localizations are in concordance with recently published data on the homologous SARS-CoV ORF 3a protein that is responsible for Golgi membrane rearrangement [42].

### Colocalization of hCoV-NL63 ORF 3 protein with structural proteins

**Figure 4:**
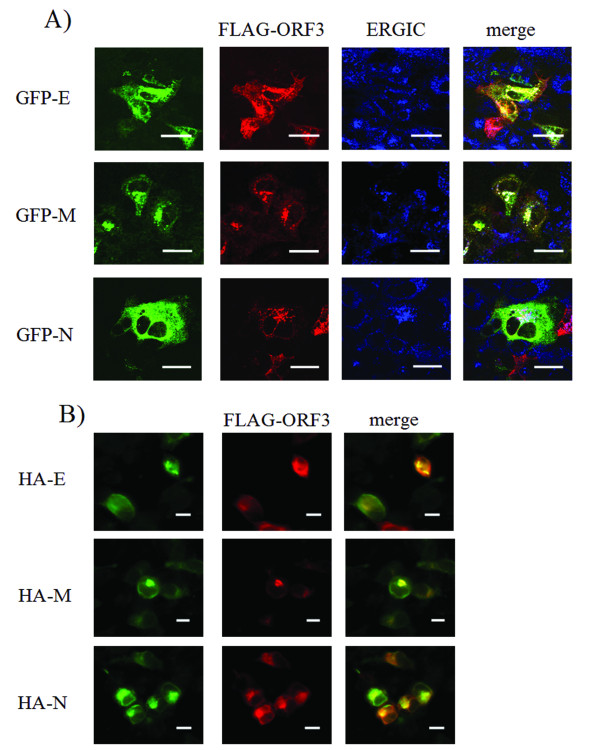
**Subcellular localization of overexpressed hCoV-NL63 proteins in Huh-7 and HEK-293T cells**. Confocal laser scanning microscopy on cells co-expressing GFP-E, GFP-M, GFP-N, respectively, together with FLAG-ORF 3. (A), Huh-7 cells. The green panels on the left show GFP fluorescence from overexpressed E, M, and N proteins. Red pictures in the next column show Cy3 fluorescence from anti-FLAG staining of overexpressed FLAG-ORF 3 fusion protein. Blue pictures show Cy5 fluorescence from staining of the ER-Golgi intermediate compartment (ERGIC) (refer to Materials and Methods section for antibodies and staining technique). Yellow areas in the right hand column represent colocalization of the GFP-proteins with FLAG-ORF 3 whereas white regions in merged pictures show colocalization of GFP proteins with FLAG-ORF 3 within the ERGIC. GFP-E and M show excessive colocalization with FLAG-ORF 3 especially within the ERGIC in both cell lines. GFP-N partially colocalizes with FLAG-ORF 3 mainly within the ERGIC. Analysis was performed with the help of a confocal laser scanning microscope (cLSM 510 Meta, Zeiss). Bars represent 20 μm. (B), to exclude altered subcellular localization contributed by the fusion tags on the overexpressed structural proteins, experiments were repeated in HEK-293T cells using FLAG-ORF 3 in combination with HA tagged E, M and N proteins. Bars represent 10 μm.

For SARS-CoV ORF 3a protein, colocalization with the structural proteins S, E, and M, but only partial colocalization with N has been suggested [36]. To investigate colocalization of NL63-ORF 3 protein with structural proteins, an expression plasmid containing ORF 3 with an N-terminal FLAG-tag epitope was co-transfected with vectors coding for green fluorescent protein (GFP) fused to hCoV-NL63 E, M and N proteins, respectively. Expression of proteins with correct molecular weights was confirmed by Western blot analysis (data not shown). The ERGIC compartment was stained in transfected cells as described above. As shown in Figure 4, GFP-E and GFP-M both showed extensive colocalization with FLAG-ORF 3 protein. Protein complexes were localized predominantly within the ERGIC, represented by white areas in Figure 4. GFP-N had primarily a cytosolic distribution but there were small areas of colocalization with FLAG-ORF 3 protein, within the ERGIC compartment. All experiments were done in Huh-7 cells supportive of hCoV-NL63 replication, but these same findings were also confirmed in another cell line, human embryonic kidney (HEK)-293T (data not shown).

To rule out altered subcellular localization contributed by the fusion tags on the overexpressed structural proteins, experiments were repeated using FLAG-ORF 3 protein in combination with HA tagged E, M and N proteins in HEK-293T cells (Figure 4B). Again, colocalization of ORF 3 protein with E and M protein and, to a far lesser extent, with N protein was seen.

### Posttranslational modification of ORF 3 protein

**Figure 5:**
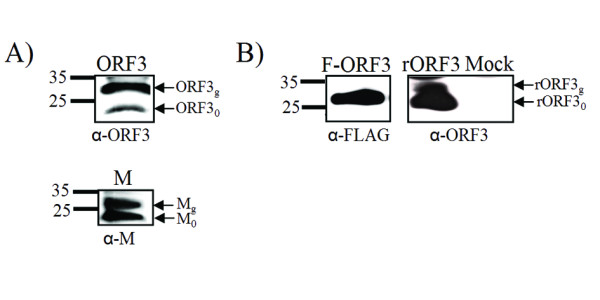
**Comparison of ORF 3 protein in viral infection and overexpression by Western blot**. (A), LLC-MK2 cells were inoculated with hCoV-NL63 (MOI 0.01) and analyzed by Western blot after 4 days using antibodies against the ORF 3 protein C-terminus (top) and against M (bottom). The bands named ORF 3_0 _and M_0 _are corresponding to the predicted molecular weights of both proteins (26 kDa). Larger bands ORF 3 g and Mg were assumed to be the result of posttranslational modification. (B, left panel): HEK-293T cells transfected with N-terminally FLAG-tagged ORF 3 do not show signs of posttranslational modification as observed in (A). (B, right panel): overexpression of ORF 3 protein in the same system without an N-terminal fusion tag reconstitutes the additional band of higher molecular weight observed in infected cells. The "mock" lane represents a control transfected with an empty vector.

Posttranslational modification of the ORF 3 protein in hCoV-NL63-infected LLC-MK2 cells was analyzed by Western blot. The M protein which had a very similar predicted molecular mass of 26 kDa (Table 1) served as a control. As expected, the M protein and a protein corresponding to ORF 3 protein migrated at corresponding heights in Western blots (Figure 5A). Both proteins showed additional bands at slightly higher molecular mass, consistent with posttranslational modification. In contrast to virus-infected cells, cells overexpressing ORF 3 protein from plasmid with an N-terminal FLAG epitope showed only a single band in Western blot whose migration was consistent with the hypothetical unglycosylated form (Figure 5B, left panel). It was assumed that glycosylation at the predicted N-glycosylation site at position 16 (Table 1) might be ablated in the overexpressed protein, due to presence of the N-terminal epitope tag. Indeed, recombinant ORF 3 (rORF 3) protein without any tag and overexpressed in the same cells from the same vector showed both forms, identical to those observed in virus-infected cells (Figure 5B, right panel). To determine whether N-terminal glycosylation was to be expected at position 16, the membrane topology of the N-terminus was examined next.

### Topology of ORF 3 protein

**Figure 6:**
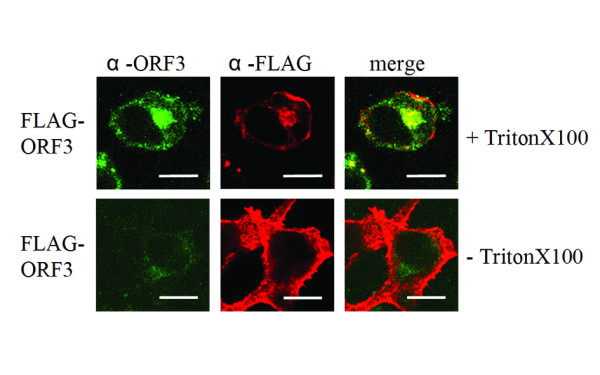
**Topology of recombinant FLAG-tagged ORF 3 protein**. Recombinant N-terminal tagged FLAG-ORF 3 protein was transiently expressed in HEK-293T cells and localization was analyzed by confocal laser scanning microscopy (cLSM 510 Meta, Zeiss). FLAG-ORF 3 protein was stained with rabbit-anti-ORF 3 recognizing the C-terminus and mouse-anti-FLAG for detection of the FLAG-tagged N-terminus (upper panel). Permeabilized cells (+TritonX100) show colocalized signals mainly in perinuclear regions for protein ORF 3 C-terminus and N-terminus whereas without permeabilization (-TritonX100) only FLAG-tagged N-terminus of protein ORF 3 could be detected at the plasma membrane (lower panel). Bars represent 10 μm.

Based on our *in-silico *analyses and in agreement with reports on SARS-CoV ORF 3a protein [36], we hypothesized that the hCoV-NL63 ORF 3 protein N-terminus reached the ER lumen and was eventually exposed on the cell surface. For confirmation, N-terminally FLAG-tagged ORF 3 protein was overexpressed in HEK-293T cells and stained by IFA using monoclonal antibodies against the FLAG tag, or alternatively, a polyclonal antibody against a peptide representing the ORF 3 protein C-terminus. As shown in Figure 6, a perinuclear distribution of fluorescence was observed with both antibodies in permeabilized cells. In non-permeabilized cells, only the anti-FLAG antibody yielded fluorescence at cell surfaces. Unfortunately, there was no complete overlap of signals from both antibodies in fully permeabilized cells in merged fluorescence pictures, most likely due to additional non-specific recognition of non-viral epitopes by the polyclonal antibody against the ORF 3 protein C-terminus. For this reason a clear intracellular localization of the C-terminus in relation to the ER/Golgi membrane could not be formally determined. However, it could be concluded that the N-terminus of the ORF 3 protein was facing towards the extracellular space.

### N-glycosylation of *in-vitro *translated ORF 3

**Figure 7:**
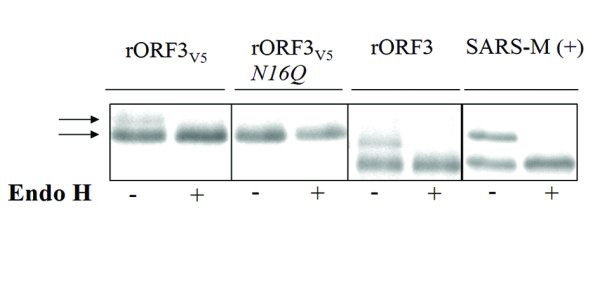
**N-glycosylation of hCoV-NL63 ORF 3 protein**. HCoV-NL63 ORF 3 protein with and without a C-terminal V5 tag, and with an N16Q exchange in the tagged version was *in-vitro *translated in presence of ^35^S-methionine. SARS-CoV M protein without a tag was translated in the same system as a control. Proteins were digested with endoglycosidase (Endo H) as indicated below each lane, subjected to SDS-PAGE, and visualized. Note the removal of the bands of increased molecular weight for the control and ORF 3 proteins, but not for the ORF 3 protein with an amino acid exchange at the hypothetical N-glycosylation site. Note also that extent of size reduction for the SARS-CoV M protein, which is known to have one N-terminal N-glycosylation site, is the same for the NL63 ORF 3 protein.

According to *in-silico *predictions the ORF 3 protein contained three putative N-glycosylation sites at positions 16, 119 and 126 (Figure 1B, Table 1). Only position 16 was considered a possible N-glycosylation target, as the other two positions would be located within the membrane. In a vector expressing ORF 3 protein with a C-terminal V5 tag, asparagine (N) at position 16 was changed into glutamine (Q). *In-vitro *translated ^35^S-radiolabelled proteins with and without the exchange were treated or not treated with endoglycosidase H prior to SDS-PAGE analysis. SARS-CoV M protein served as the control because it had been shown previously to be N-glycosylated exclusively at position four [34]. *In-vitro *translated NL63 protein ORF 3 with and without the V5 tag, but not the same protein with an N16Q exchange, showed a second band of increased molecular weight in SDS-PAGE that disappeared upon endoglycosidase H treatment (Figure 7). In the same way as for SARS-CoV M-protein, deglycosylation did not change the apparent molecular weight of the lower band, verifying absence of any further active glycosylation sites.

### NL63-ORF 3 protein is a structural viral protein

**Figure 8:**
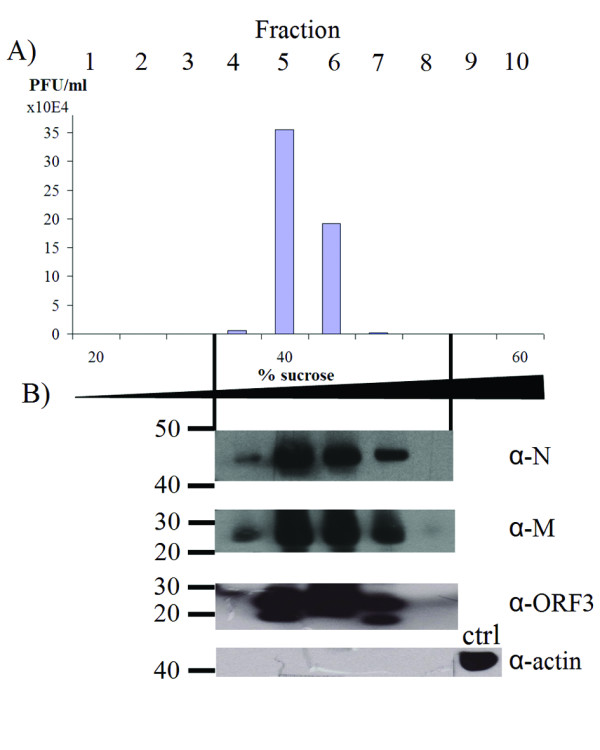
**Identification of NL63-ORF 3 protein as a structural viral protein by sucrose gradient ultracentrifugation**. Viral supernatant was purified via subsequently centrifugation on two discontinuous and one continuous sucrose gradients of 20% to 60% (w/v) sucrose. The continuous cushion was divided into ten fractions as indicated in part (A). After centrifugation of each fraction through 20% sucrose cushions, the resulting pellets were analyzed for infectious particles by plaque assays. Resulting virus titers are indicated on the 20 Y-axis in part (A). (B), fractions 4-8 were subjected to Western blot analysis using specific rabbit antibodies against ORF 3, M and N protein (1:3000; 1:250,000 and 1:24,000, respectively). To exclude cellular contaminations in the fractions a Western blot using mouse-anti-actin (1:2,000) was performed. Note the colocalization of the ORF 3 protein in the same gradients as the known structural proteins M and N.

Our data suggested that the ORF 3 protein was a glycosylated protein that colocalized with structural proteins in the ERGIC. Protein ORF 3 might thus constitute a structural protein itself. To assess if the ORF 3 protein was incorporated into virions, viral particles were purified by sucrose gradient ultracentrifugation. After centrifugation, the gradient was divided into ten fractions and infectivity within each fraction was determined by plaque assay (Figure 8). Only fractions 4 to 7 correlating with a sucrose density of 35% to 45% contained infectious particles with a peak of 3.6 × 10E5 PFU/ml in fraction 5 (sucrose density 40-41%). Subsequent Western blot analysis identified the same pattern of accumulation within the gradient for the ORF 3 protein as for the structural M and N proteins. Anti-actin staining excluded cellular contamination in these fractions. It was concluded that hCoV-NL63 ORF 3 protein was incorporated into viral particles.

## Conclusions

The ORF 3 protein and its homologues are conserved among CoVs [30]. Although identities on nt and aa level are low, most are predicted to be triple membrane-spanning proteins [35]. While it has been suggested that ORF 3 homologues are dispensable for replication in cell culture, mutations of ORF 3 homologues in transmissible gastroenteritis virus (TGEV) and porcine epidemic diarrhea virus (PEDV) lead to attenuation of virus *in-vivo *in pig models [44,51,52]. Because the SARS-ORF 3a protein underwent positive selective pressure during the human epidemic in 2002/2003 [53], an important function *in-vivo *can be assumed for the SARS-CoV ORF 3a protein as well.

Unfortunately, it remains difficult to characterize *in-vivo *functions of hCoV-NL63 ORF 3 protein due to lack of any animal model. However, it is interesting to note that across all strains of hCoV-NL63 characterized so far, there are no mutations in the ORF 3 amino acid sequence [46,54]. Conservation of ORF 3 matches results by Donaldson et al., showing that virus production in human airway epithelium was reduced when the ORF 3 protein was replaced by GFP [28,46]. It has thus been suggested that protein ORF 3 might serve functions involved in viral egress which are relevant for spreading in airway epithelium but not in simpler cell culture [46].

Results from this study, in particular the subcellular localization of ORF 3 protein along the secretory pathway (ERGIC, Golgi, plasma membrane), the colocalization of NL63-ORF 3 protein with other structural proteins in the ERGIC and the inclusion of the ORF 3 protein in virions give support for a hypothetical function within the viral assembly and budding process. A range of further hypotheses can be derived from earlier investigations into protein ORF 3 functions. These include antigen decoy functions as suggested for SARS-CoV ORF 3a [55], interference with the regulation of expression of NFκB-dependent cytokines [56,57] and fibrinogen [39], and finally the modulation of S protein mediated endocytosis [36] or an hypothesized down-regulation of the expression of S protein on the cell surface [58].

## Materials and methods

### Cell culture and materials

Rhesus monkey kidney LLC-MK2 cells (ATCC: CCL-7), human embryonic kidney HEK-293T cells (ATTC: CRL-1573), human hepatocellular carcinoma cell line (Huh-7, JCRB0403 kindly provided by Antoine A. F. de Vries, LUMC, Leiden) and colon carcinoma CaCo-2 cells (ATCC: HTB-37) were grown at 37°C and 5% CO_2 _in Dulbecco's Modified Eagles Medium (DMEM; Gibco, Karlsruhe, Germany) containing 10% fetal calf serum, 2 mM L-glutamine and 25 U of penicillin/ml and 25 U streptomycin/ml (PAA Laboratories, Linz, Austria). All cells were tested negative for mycoplasms by PCR as described elsewhere [59]. If not stated otherwise materials were provided from Roth, Karlsruhe, Germany.

### Virus infections with hCoV-NL63 and plaque assay

For virus stock production either CaCo-2 or LLC-MK2 cells were inoculated with hCoV-NL63 (8^th ^passage Amsterdam strain I; accession no. NC_005831) at a multiplicity of infection (MOI) of 0.01 and infected cells were cultured at 37°C and 5% CO_2 _for five to seven days before harvesting. After centrifugation at 6,000 × g for 10 min supernatant was aliquoted and stored at -80°C. Titers were determined by plaque assay performed as described elsewhere [60]. Briefly, after incubation of the plaque assays at 37°C and 5% CO_2 _for four days cells were fixed with 4% formaldehyde, stained with crystal violet solution and results were interpreted as described previously [61].

### Construction of plasmids

For first strand cDNA synthesis total RNA was extracted from infected cells five to seven days post infection (dpi). Reverse transcription was performed as described elsewhere [62] using oligo(dT) primers (Fermentas, St. Leon-Roth, Germany). In order to recombinantly express hCoV-NL63 proteins ORF 3, E, M and N we cloned the different genes into a variety of expression vectors. For generation of GFP-constructs PCR was performed with the following specific primers listed in Table 2: E: 5'NL63-E-GFP and 3'NL63-EpK R, M: 5'NL63-M-GFP and 3'NL63-MpK R, N: 5'NL63-N-GFP and 3'NL63-NpK R, ORF 3: 5'NL63-O3-GFP and 3'NL63-O3. For producing the pcDNA3.1-ORF 3-V5/His construct which was used for in-vitro translation experiments we applied primers 5'Leader-NL and 3'NL-O3s. Mutagenesis for the N16Q construct was done with primers NL63-O3mis-Asn16 F and R using QuickChange Mutagenesis kit (Stratagene/Agilent Technologies, Waldbronn, Germany) according to the manufacturer's instructions.

**Table 2:** Oligonucleotides^a ^used for cloning procedures

Primer	Sequence (5'-end to 3'-end)		+/-	NC_005831^b^
pcDNA3.1/NT-GFP-TOPO				
5'NL63-E-GFP	TTCCTTCGATTAATTGATGAC		+	25203-25223
5'NL63-M-GFP	TCTAATAGTAGTGTGCCTC		+	25445-25463
5'NL63-N-GFP	GCTAGTGTAAATTGGGCC		+	26136-26153
5'NL63-O3-GFP	CCTTTTGGTGGCCTATTTC		+	24545-24563
3'NL63-EpK R	TTAGACATTTAGTACTTCAGCTGG		-	25410-25433
3'NL63-MpK R	TTAGATTAAATGAAGCAACTTCTC		-	26099-26122
3'NL63-NpK R	TTAATGCAAAACCTCGTTGAC		-	27246-27266
3'NL63-O3	ACAAGGAGCCATAAAATG		-	25244-25261
				
pcDNA3.1/V5-His-TOPO				
5'Leader-NL	GACTTTGTGTCTACTCTTC		+	45 - 63
3'NL63-O3s	ATTAATCGAAGGAACATC		-	25199-25216
NL63-O3mis-Asn16 F	CTTACTCTTGAAAGTACTATTCAGAAGAGTGTGGCTAATCTC		+	25567-25609
NL63-O3mis-Asn16 R	GAGATTAGCCACACTCTTCTGAATAGTACTTTCAAGAGTAAG		-	25567-25609
				
pCAGGS^c^				
5'Eco-FLAG_O3-63	GCAGCAGAATTC*ATGGACTACAAGGACGACGATGACAAG*CCTTTTGGTGGCCTATTTCAACTTAC		+	24544-24570
3'Not-O3-63	CCTCCTGCGGCCGCTCAATTAATCGAAGGAACATCTTCGTATAG		-	25190-25219
5'Eco-HA-E	GCAGCAGAATTC*ATGTACCCATACGATGTTCCAGATTACGCT*TTCCTTCGATTAATTGATGACAATG		+	25203-25227
3'Not-E	CCTCCTGCGGCCGCTTAGACATTTAGTACTTCAGCTG		-	25411-25433
5'Eco-HA-M	GCAGCAGAATTC*ATGTACCCATACGATGTTCCAGATTACGCT*TCTAATAGTAGTGTGCCTCTTTTAGAG		+	25446-25472
3'Not-M	CCTCCTGCGGCCGCTTAGATTAAATGAAGCAACTTCTCTC		-	26098-26123
5'Eco-HA-N	GCAGCAGAATTC*ATGTACCCATACGATGTTCCAGATTACGCT*GCTAGTGTAAATTGGGCCGATGACAG		+	26138-26163
3'Not-N	CCTCCTGCGGCCGCTTAATGCAAAACCTCGTTGACAATTTC		-	27242-27268

^a^Oligonucleotides were provided by TIBMolbiol, Berlin, Germany

^b^Accession no. hCoV-NL63 strain Amsterdam I

^c^Underlined are additional nucleotides representing restriction sites and a FLAG or HA-tag (italics)

For PCR amplification of FLAG-ORF 3 as well as HA tagged E, M and N and subsequent cloning into a pCAGGS vector (kindly provided by Prof. Dr. Stephan Becker, University of Marburg) we used 5'Eco-FLAG_O3-63 and 3'Not-O3-63, 5'Eco-HA-E and 3'Not-E, 5'Eco-HA-M and 3'Not-M, 5'Eco-HA-N and 3'Not-N, respectively (Table 2). In this case PCR products were digested with restriction endonucleases EcoRI and NotI (Fermentas) before cloning into the pCAGGS vector (also digested and additionally dephosphorylated before use).

Generally, PCR was performed with Platinum^® ^*Taq *DNA Polymerase High Fidelity (Invitrogen, Karlsruhe, Germany), and conditions were as follows: 94°C for 2 min, followed by 35 cycles of 94°C for 30 s, primer specific temperature for 30 s, and 72°C for 90 s, with a final extension at 72°C for 10 min. The different genes were cloned into pcDNA3.1/V5-His-TOPO (eukaryotic expression and *in-vitro *translation) and pcDNA3.1/NT-GFP-TOPO (eukaryotic expression) with the help of TOPO Expression Kits (Invitrogen) according to the manufacturer's instructions. Cloning of FLAG-tagged ORF 3 into the pCAGGS vector was done conventionally with T4 ligase (Invitrogen) according to suppliers' description. Correct cloning was confirmed by sequencing (Abi Prism 3,100; Applied Biosystems, Foster City, USA).

### Generation of polyclonal ORF 3 antiserum

The generation of a polyclonal antiserum against ORF 3 was done with the help of keyhole limpet hemocyanin (KLH) coupled peptides. Two peptides were synthesized corresponding to aa positions 182-197 and 211-225 (Eurogentec, Seraing, Belgium). Immunization was performed in-house. Briefly, a chinchilla rabbit was immunized four times with 200 μg of a mixture of the two KLH coupled peptides and sera were tested as suggested by the manufacturer by enzyme-linked immunosorbent assay (ELISA) using the corresponding uncoupled peptides. We then tested serum with IFA using infected LLC-MK2 cells (Figure 2) as well as with prokaryotic recombinant proteins with the help of Dot blot and Western blot analysis (data not shown). The bleeding for the applied anti-ORF 3 serum was carried out 20 days after the fourth injection and sera were used directly.

### Expression analysis and subcellular localization studies of native viral proteins by indirect IFA and Western blot

Typically, 8 × 10^4 ^CaCo-2 or LLC-MK2 cells were seeded on glass slides in a 24-well plate and infected with hCoV-NL63 as described above. Two to four days after infection the cells were fixed with paraformaldehyde (4%) for 15 min and permeabilized with 0.1% TritonX100 (Merck, Darmstadt, Germany) for 10 min. Afterwards the cells were washed with PBS again and then incubated with the primary antibody, diluted 1:100 in sample buffer (EUROIMMUN, Lübeck, Germany), at 37°C for 1 h. The ERGIC was stained with the help of mouse-anti-ERGIC53 (Axxora, Grünberg, Germany). In order to stain the Golgi apparatus we used a mouse-anti-Golgi 58 K (Sigma-Aldrich, Munich, Germany). For staining of the *trans*-Golgi Network and lysosomal compartment we applied a goat-anti-LAMP-1 antibody (Santa Cruz Biotechnology, Heidelberg, Germany). Secondary detection was done with fluorescein isothiocyanate (FITC) or cyanine 2 (Cy2)-conjugated goat-anti-rabbit as well as with rhodamine or Cy3-conjugated goat-anti-mouse or donkey-anti-goat antibody (Dianova, Hamburg, Germany) at 37°C in a wet chamber for 30 min. Slides were mounted and analyzed by cLSM 510 META laser confocal microscope (Zeiss, Jena, Germany).

Western blot analysis of viral proteins was done as described elsewhere [63]. For titration of the different rabbit antisera we used hCoV-NL63 cell lysate generated from LLC-MK2 infected cells five to seven dpi (~1 × 10^7 ^cells/blot) for Western blotting and incubated the produced nitrocellulose strips with the different rabbit antisera (pre-immune sera as negative control) at dilutions ranging from 1:500 up to 1:256,000 (data not shown). Generally, cells were lysed in RIPA lysis buffer (150 mM NaCl, 1% Igepal CA-630, 0.5% sodium deoxycholat, 0.1% SDS, 50 mM Tris (pH 8.0)) and separated on a 12% SDS-PAGE gel. Western blotting was performed by using anti-ORF 3, anti-M, anti-N at dilutions 1:4,000, 1:250,000 and 1:24,000 respectively. Secondary detection was done with the help of SuperSignal^® ^West Dura Extended or Femto Chemiluminescence Substrate (Pierce Biotechnology, Rockford, USA).

### Transient transfection of recombinant proteins for colocalization studies by indirect IFA and Western blot analysis

Transfections of HEK-293T and Huh-7 cells with eukaryotic expression vectors containing the fusion genes GFP-E, GFP-M, GFP-N, HA-E, HA-M, HA-N and FLAG-ORF 3 were performed with the help of FuGENE HD (Roche, Basel, Switzerland) transfection reagent as described above using 24-well plates provided with glass slides. After a 24 h incubation at 37°C and 5% CO_2_transfected cells were washed with PBS and fixed with paraformaldehyde (4%), permeabilized with TritonX100 and incubated with rabbit-anti-FLAG (Sigma) and mouse-anti-ERGIC53 (Axxora) primary antibodies, both diluted 1:100 with sample buffer (EUROIMMUN). Secondary detection was performed with Cy3-conjugated goat-anti-rabbit (1:200) and Cy5 labelled goat-anti-mouse (1:100) antibodies (Dianova). Slides were mounted and analyzed by confocal laser scanning microscopy. For Western blot analysis of recombinant ORF 3 proteins (FLAG-ORF 3, rORF 3) transfections were performed in 6-well plates using FuGENE HD transfection reagent. Transfection was performed with 6 μg DNA and 12 μl FuGENE HD in 100 μl DMEM. Transfected cells were washed three times with ice cold PBS and harvested for Western blot analysis after incubation for 26 to 48 h at 37°C and 5% CO_2_. Cell lysis was performed with RIPA lysis buffer (~4 × 10^7 ^cells/ml) containing Protease Inhibitor Cocktail III (Calbiochem, San Diego, USA) and Benzonase (25 U/ml) (Novagen, Madison, USA). After 30 min incubation on ice samples were sonicated twice for 30 s (Branson Sonifier 450, Branson, Danbury, USA) and centrifuged at 13,000 × g for 1 min at 4°C. For detection of the different proteins we used rabbit-anti-FLAG (Sigma, diluted 1:5,000) or anti-ORF 3 antiserum (1:3000) and incubated blots for 1 to 2 h at room temperature. As secondary antibody we applied a goat-anti-mouse or rabbit horseradish peroxidase (HRP)-conjugated antibody (Pierce Biotechnology) for 1 h at room temperature. Detection was performed by using SuperSignal^® ^West Femto Chemiluminescence Substrate (Pierce Biotechnology).

### *In-vitro *translation of ORF 3 and analysis of glycosylation by endoglycosidase H digestion

Plasmids pcDNA3.1-ORF 3-V5/His, pcDNA3.1-ORF 3-N16Q-V5/His and pcDNA3.1-ORF 3 were employed in the TNT T7 quick coupled reticulocyte lysate system (Promega, Mannheim, Germany) according to the manufacturer's description. The proteins were metabolically labelled with [^35^S]methionine (GE Healthcare, Munich, Germany) and translated in the presence of canine pancreatic microsomal membranes (Promega). Membrane-bound proteins were pelleted at 13,000 × g for 15 min and resuspended in PBS. Samples were split in half and incubated for 1 h at 37°C with endoglycosidase H (Endo H; New England Biolabs, Frankfurt, Germany) or, as control, without additives. Afterwards samples were subjected to SDS-PAGE. Radioactive signals were visualized by exposing dried gels to BioImage plates, which were scanned by using a bioimager analyzer (BAS-1,000; Fuji).

### Purification of viral particles by sucrose gradient ultracentrifugation

Purification of viral particles was performed by sucrose gradient ultracentrifugation as described elsewhere [33]. Briefly, 45 ml viral supernatant from infected CaCo-2 cells was cleared from cell debris 4 dpi and subsequently applied onto two discontinuous and one continuous sucrose cushion of 20% to 60%. The continuous cushion was divided into ten fractions and viral particles were pelleted by ultracentrifugation through a 20% sucrose cushion. Virus pellets were resuspended in 100 μL PBS and stored at -80°C.

### *In-silico *analyses

Prediction of protein topology and subcellular localization was done by NetNGlyc, NetOGlyc, TMHMM http://www.cbs.dtu.dk/services/, TMPred http://www.ch.embnet.org/software/TMPRED_form.html, and ProDiV/TOPCONS http://topcons.cbr.su.se/index.php. The alignments and a sequence identity matrix were done by using BLAST and MEGA4 (BLOSUM; parameters p-distance and pair wise deletion).

## Competing interests

The authors declare that they have no competing interests.

## Authors' contributions

MAM, MN, CD conceived and performed the experiments DV, OB, DL, ARS, SK, TS, LvdH, BCF; assisted in experiments and contributed reagents. MAM; CD wrote the manuscript. All authors have read and approved the final manuscript.
